# Dexamethasone can potentiate the anti-emetic action of a 5HT3 receptor antagonist on cyclophosphamide induced vomiting in the ferret.

**DOI:** 10.1038/bjc.1990.12

**Published:** 1990-01

**Authors:** J. Hawthorn, D. Cunningham

**Affiliations:** Department of Physiology, St Georges Hospital Medical School, London, UK.

## Abstract

A new group of selective 5HT3 antagonists are proving to be effective anti-emetics for cytotoxic and radiation induced vomiting in both animal models and man. Current anti-emetic regimens often benefit from combination therapy, in particular the efficacy of metoclopramide (which can be a weak 5HT3 antagonist), can be improved by combination with dexamethasone, another anti-emetic. Hence it was of interest to evaluate whether a 5HT3 receptor antagonist GR38032F could be improved by combination with dexamethasone. Vomiting induced by cyclophosphamide in the ferret was observed after pre-treatment with dexamethasone alone or in combination with GR38032F. Animals were also observed for signs of 'nausea'. Dexamethasone alone proved a weak anti-emetic in this system but did have significant effects on 'nausea'. GR38032F has previously been shown to be capable of totally controlling emesis due to cyclophosphamide in the ferret. Here a dose of GR38032F that is not 100% effective was employed; this was shown to have effects on 'nausea' but most interestingly its anti-emetic action was increased by combination with dexamethasone. This may be important for the minority of patients whose vomiting is not completely controlled by GR38032F alone.


Br. J. Cancer (1990), 61, 56-60 © Macmillan Press Ltd., 1990

### Dexamethasone can potentiate the anti-emetic action of a $5HT_3$ receptor antagonist on cyclophosphamide induced vomiting in the ferret

J. Hawthorn[1] & D. Cunningham[2]

[1]*Department of Physiology, St Georges Hospital Medical School, London SW17 0RE, UK; and* [2]*Section of Medicine, Institute of Cancer Research, Royal Marsden Hospital, Downs Road, Sutton, Surrey SM2 5PT, UK.*

**Summary** A new group of selective $5HT_3$ antagonists are proving to be effective anti-emetics for cytotoxic and radiation induced vomiting in both animal models and man. Current anti-emetic regimens often benefit from combination therapy, in particular the efficacy of metoclopramide (which can be a weak $5HT_3$ antagonist), can be improved by combination with dexamethasone, another anti-emetic. Hence it was of interest to evaluate whether a $5HT_3$ receptor antagonist GR38032F could be improved by combination with dexamethasone. Vomiting induced by cyclophosphamide in the ferret was observed after pre-treatment with dexamethasone alone or in combination with GR38032F. Animals were also observed for signs of 'nausea'. Dexamethasone alone proved a weak anti-emetic in this system but did have significant effects on 'nausea'. GR38032F has previously been shown to be capable of totally controlling emesis due to cyclophosphamide in the ferret. Here a dose of GR38032F that is not 100% effective was employed; this was shown to have effects on 'nausea' but most interestingly its anti-emetic action was increased by combination with dexamethasone. This may be important for the minority of patients whose vomiting is not completely controlled by GR38032F alone.

Nausea and emesis are the most distressing side-effects of cancer chemotherapy (Coates *et al.*, 1983), causing anxiety, demoralisation of the patient and, in extreme cases, patient non-compliance (Laszlo, 1981). The actual trigger(s) causing this nausea and vomiting is complex, probably involving the direct effects of the drugs, their metabolites or substances which they release at both peripheral and central sites (for review see Andrews & Hawthorn, 1988).

One of the most effective and widely used anti-emetics is metoclopramide (for review see Gralla, 1983). Many anti-emetic regimens benefit from combination therapy and in this context the action of metoclopramide is enhanced by the concomitant administration of dexamethasone (Bruera *et al.*, 1982; Allan *et al.*, 1984; Palmer & Colls, 1987). However, even this combination is far from totally effective. Following the demonstration that metoclopramide was more effective at high doses (Gralla *et al.*, 1981) and that high dose metoclopramide showed antagonism at the $5HT_3$ (or M) type receptor (Fozard, 1984) a new group of selective $5HT_3$ receptor antagonists, then recently developed (Brittain *et al.*, 1987; Fake *et al.*, 1987; Fozard, 1984; Richardson *et al.*, 1985) were investigated as possible anti-emetics. Their effectiveness has surpassed other anti-emetic therapies in both animal models (Miner & Sanger, 1986; Miner, Sanger & Turner, 1987; Hawthorn *et al.*, 1988; Costall *et al.*, 1986; Stables *et al.*, 1987; Bermudez *et al.*, 1988) and human studies (Cunningham *et al.*, 1988; Liebundgut *et al.*, 1988; Carmichael *et al.*, 1988). So far they have only been employed singly and hence it was of interest to evaluate whether the effect of a $5HT_3$ receptor antagonist, GR38032F (Glaxo), against cyclophosphamide-induced vomiting in the ferret could be improved by combination with dexamethasone. The ferret is an animal that is well established for studies of emesis (Florczyck *et al.*, 1982; Gylys & Gidda, 1986; Hawthorn *et al.*, 1988; King, 1988; Tuor *et al.*, 1988) shows behavioural perturbations which may indicate nausea (Bermudez *et al.*, 1987; Hawthorn & Andrews, 1988) and is a good predictive model of anti-emetics for man (Miner *et al.*, 1987). We employed a dose of GR38032F that had been established as effective in delaying but not reducing the emesis in this model (Stables *et al.*, 1987) and observed whether this was influenced by dexamethasone. We also studied the effect of dexamethasone alone. As this anti-emetic has not been assessed in the ferret before, this would further extend our knowledge of the ferret as a suitable model for anti-emetic studies.

Correspondence: D. Cunningham.
Received 22 May 1989; and in revised form 18 August 1989.

### Materials and methods

### *Animals and drugs*

The animals used were albino or fitch ferrets (*Mustela putorius furo* L.) of either sex weighing between 500 and 1,200 g. They were housed singly under a 12-h light cycle and fed *ad libitum* on a standard carnivore diet. Food was withdrawn the night before experimentation. The following morning they were placed in a clear perspex observation pen (70 cm × 40 cm × 60 cm) and filmed on video tape in the absence of any observers. After a 40 min period the appropriate pre-treatment of GR38032F (Glaxo) or dexamethasone (Oradexon, Organon) was administered subcutaneously into the shoulder. GR38032F was dissolved in 154 mM NaCl, dexamethasone was supplied as the sodium phosphate in aqueous solution. The total injection volume was $<1.0$ ml.

The animals were returned to the observation pen and given milk to drink *ad libitum*; this facilitated subsequent observation of emesis. Filming continued for a further 30-40 min, after which they were injected intra-peritoneally with 200 mg $kg^{-1}$ cyclophosphamide; this is the $ED_{100}$ (in terms of producing emesis) in this species (Hawthorn *et al.*, 1988). Cyclophosphamide (Sigma) was dissolved in alcohol (1 mg $ml^{-1}$) and diluted with 154 mM NaCl. The final injection volume was 2-3 ml. Filming was continued for a further 4 h after which the animals were killed by an overdose of pentobarbitone (Euthetal, May and Baker).

The groups investigated were controls, GR38032F 0.1 mg $kg^{-1}$ alone, dexamethasone at 2 mg $kg^{-1}$ or 5 mg $kg^{-1}$ and combinations of GR38032F 0.1 mg $kg^{-1}$ plus dexamethasone 2 mg $kg^{-1}$ or GR38032F 0.1 mg $kg^{-1}$ plus dexamethasone 5 mg $kg^{-1}$.

### *Analysis of video tapes*

Later analyses of the video tapes quantified the number of retches and vomits and the times at which they occurred. The behaviour of the animals was assessed on a points system as described previously (Hawthorn & Andrews, 1988) and briefly outlined here. After considerable time analysing video tapes and timing the number of occasions on which behaviour occurred we were able to draw up a points system for rating nausea. A point was awarded for the presence of 'nausea-related' behaviour and for the absence of behaviour inhibited by 'nausea'. The positive behaviours were: slit eyed appearance, licking, lying with the chin down on the floor, running backwards, burrowing, walking on tiptoes, lying totally prostrate with hind limbs plantar flexed, assuming a posture like a recumbent 'S', walking while dragging their belly along the ground, pressing the nose up against the side of the pen, holding a very still position with the nose pointing up in the air, being unable to sleep comfortably and falling over. The behaviours scored for their absence were: standing on hind legs, grooming, rolling over, sniffing and playing with a drinking bowl. Using this system 'nausea' could be reliably and reproducibly scored in ferrets which had received emetic agents.

Control behaviour was assessed during the time period 20–40 min after the animals were placed in the observation pen. This allowed a 20 min initial period for the animal to become familiar with the pen. The 20 min period immediately following pre-treatment was used to assess the behavioural effects of GR38032F or dexamethasone. After administration of cyclophosphamide the first 20 min period was not analysed and the observations were made at 20–40 min, as 20 min corresponds to the latency for cyclophosphamide to induce emesis and the associated behavioural changes in this species.

The patterns of retching and vomiting were obtained by counting the number of retches or vomits that occurred in each 10 min time interval following administration of the cyclophosphamide, and taking an average of each 10 min 'bin' across groups. Total retches and vomits are quoted for the entire 4 h observation period. Values are given as the mean ± s.e.m. Even though the same animals were used for sequential observations the combination of data from various groups has necessitated the use of an unpaired *t* test.

### Results

The amount of retching and vomiting in response to cyclophosphamide with or without anti-emetic pre-treatment is given in Table I. Control animals did not start to retch or vomit until 18.0 ± 3.2 (mean ± standard error) min after administration of the drug. GR38032F at 0.1 mg kg$^{-1}$ delayed the onset of retching and vomiting to 99.0 ± 3.2 and 121.2 ± 30.0 min respectively. The increases in latency were highly significant ($P<0.005$ and $P<0.0005$), although there was no significant reduction in the total number of retches or vomits over the 4 h observation period. The duration of action of this dose of GR38032F for complete inhibition of the retching and vomiting was 140 and 160 min respectively.

The lower dose of dexamethasone (2 mg kg$^{-1}$) tended to increase the number of retches and vomits and decrease the latency however this was not significant. The higher dose (5 mg kg$^{-1}$) also paradoxically reduced the latency to retch and vomit significantly ($P<0.05$) and an initial early phase of vomiting was noted, although the total retches and vomits were reduced.

When the low dose of dexamethasone was administered with the GR38032F it had no apparent additional effect and the values obtained were very similar to those observed with GR38032F alone, showing the increased latency and only marginally decreased number of retches and vomits. In fact, the presence of GR38032F seemed to counteract the tendency for dexamethasone to decrease the latency and increase the amount of vomiting.

The higher dose of dexamethasone had a marked effect. Four animals were used in this group and one was completely protected, showing no retching or vomiting at all during the 4 h of observation. A second animal was protected for a considerable time and only had 15 retches and one vomit at 225 min. The other two animals both vomited but the total vomits (4.5 ± 2.4) were significantly reduced compared to the controls ($P<0.05$).

The patterns of retching and vomiting are shown in Figures 1 and 2. By viewing the retches and vomits occurring in 10 min time intervals the effects of the various drug pretreatments are more easily appreciated. Thus the ability of GR38032F at this low dose to delay, but not diminish, the retching and vomiting is quite clear. Dexamethasone at the high dose had a pronounced effect on the later stage of retching and especially vomiting. However, the effect of a combination of GR38032F at 0.1 mg kg$^{-1}$ and dexamethasone at 5 mg kg$^{-1}$ was the most dramatic.

Table II shows the effects of the various drug treatments on the 'nausea' experienced by the animals. The group receiving GR38032F plus the low dose of dexamethasone had not been filmed on video and thus nausea scores cannot be given for this group.

GR38032F (0.1 mg kg$^{-1}$) had a marked effect on the nausea scores at 20 min after injection of cyclophosphamide (5.5 ± 0.9 compared to controls of 12.6 ± 0.6). Part of this action is related to the ability of GR to delay the onset of emesis, as when the animals were evaluated at 100 min during periods of active emesis the nausea score was higher (7.0 ± 0.9), although this was still significantly reduced compared to controls at 20 min ($P<0.001$).

Dexamethasone also markedly reduced nausea scores and this was dose related. These scores were measured at 20 min as the latency to vomit was not appreciably altered compared to controls and it is interesting to note that the scores are reduced even though the animals were vomiting. The most marked improvement in nausea was obtained with the combination of GR38032F and the high dose of dexamethasone compared to the controls both at 20 min ($P<0.0001$) and 100 min ($P<0.001$). Although the 'nausea' score had increased by 100 min it was not significantly higher than at 20 min.

**Table I** Retching and vomiting in response to cyclophosphamide in the presence of GR38032 and/or dexamethasone

| | *Total retches* | *Total vomits* | *Latency retch* | *Latency vomit* |
|---|---|---|---|---|
| Control ($n = 8$) | 95.4 ± 30.2 | 15.6 ± 3.0 | 18.0 ± 3.2 | 18.0 ± 3.2 |
| GR 0.1 mg kg$^{-1}$ ($n = 4$) | 86.7 ± 30.8 | 12.5 ± 3.5 | 99.0 ± 31.1[b] | 121.2 ± 30.0[b] |
| Dex 2 mg kg$^{-1}$ ($n = 7$) | 101.6 ± 29.4 | 21.0 ± 4.3 | 14.7 ± 3.5 | 14.4 ± 3.5 |
| GR 0.1 mg kg$^{-1}$ Dex 2 mg kg$^{-1}$ ($n = 4$) | 74.0 ± 33.7 | 12.2 ± 5.2 | 108.0 ± 53.7[a,c] | 108.0 ± 53.7[a] |
| Dex 5 mg kg$^{-1}$ ($n = 4$) | 86.0 ± 26.0 | 10.0 ± 3.3 | 7.6 ± 1.2[a] | 8.5 ± 1.8 |
| Dex 5 mg kg$^{-1}$ GR 0.1 mg kg$^{-1}$ | 33.5 ± 15.5 (4) | 4.5 ± 2.4[a] (4) | 84.7 ± 70.6 (3) | 84.9 ± 60.6 (3) |

[a]$P<0.05$ compared to control. [b]$P<0.0005$ compared to control.
[c]$P<0.05$ compared to Dex alone.

**Figure 1** Retching in response to cyclophosphamide 200 mg kg$^{-1}$ i.p. alone or after pretreatment with varying doses of GR38032F or dexamethasone. The anti-emetics were given alone or in combination as shown on the graphs. Results are plotted as mean ± s.e.m. for the number of retches in each 10 min of the total observation period. The number of animals in each group is given in Table I.

**Figure 2** Vomiting in response to cyclophosphamide 200 mg kg$^{-1}$ i.p. Details are as in Figure 1.

**Table II** Nausea scores produced by cyclophosphamide after various anti-emetic treatments

| | *Control* | *Plus cyclo at 20–40 min* | *Plus cyclo at 100–120 min* |
|---|---|---|---|
| No treatment | 3.1 ± 0.3[c] (15) | 12.6 ± 0.6 (7) | |
| GR 0.1 mg kg$^{-1}$ | 3.25 ± 1.1 (4) | 5.5 ± 0.9[c] (4) | 7.0 ± 0.9[b] (4) |
| Dex 2 mg kg$^{-1}$ | 3.4 ± 0.6 (5) | 10.2 ± 0.7[a] (5) | |
| Dex 5 mg kg$^{-1}$ | 27.5 ± 0.25 (4) | 5.2 ± 1.0[b] (4) | |
| GR/Dex 5 mg kg$^{-1}$ | 2.25 ± 0.5 (4) | 4.5 ± 0.5[c] (4) | 6.7 ± 1.2[b] (4) |

[a]$P < 0.05$ compared to cyclo alone. [b]$P < 0.001$ compared to cyclo alone. [c]$P < 0.0001$ compared to cyclo alone.

### Discussion

In this study, in the ferret, we have shown that dexamethasone alone is a poor anti-emetic but has dose related actions on 'nausea' and that a high dose of dexamethasone is capable of potentiating the action of a sub-optimal dose of GR38032F. Dexamethasone alone had no significant effect on retching and vomiting but caused a decrease in the latency which was significant and dose related. However, 'nausea' appeared to be reduced. Nausea is a subjective experience which cannot be extrapolated directly from man to animal models but considerable information can be derived from observing animal behaviour associated with emesis. Experience has shown quite reproducible patterns of behaviour in animal models (Bermudez *et al.*, 1988; Hawthorn & Andrews, 1988) and we have previously demonstrated that assigning a 'score' to behaviour gives a good index of the discomfort or 'nausea' experienced by the animal, which responds to anti-emetic treatment (Hawthorn & Andrews, 1988).

In this study GR38032F was effective in reducing the nausea experienced 20 min after cyclophosphamide. Part of this effect was related to its action in delaying emesis, but it also produced a marked effect on nausea even when the animals were actively vomiting. This action of dexamethasone was dose related, which is important bearing in mind that the choice of dose of dexamethasone in a clinical situtation is empirical and few dose response studies have been made. The combination of dexamethasone and GR38032F reduced nausea even further, although the differences between GR38032F alone, at 20 min, dexamethasone alone and the combination therapy were small. Dexamethasone is known to produce 'feelings of well being' in man and our results have been paralleled in the clinical situation where it has been shown that the inclusion of dexamethasone with lorazepam/metoclopramide combination increased the number of patients receiving cisplatinum who were free of nausea from 16 to 50% despite only a small reduction in median number of vomiting episodes from 7.6 to 6.1 (Palmer & Colls, 1987).

In contrast to the weak activity of dexamethasone to enhance the action of GR38032F on nausea, its ability to potentiate the antiemetic activity of GR38032F was pronounced, and exceeded what could be expected if the two drugs were merely additive. Thus GR38032F alone caused a 10% reduction in retches and a 20% reduction in vomits, dexamethasone caused a similar reduction in retches and a 35% decrease in vomits, but the combination therapy produced a 65% reduction in retching and 72% reduction in vomiting.

How dexamethasone acts is unclear. It is well established that it acts at the hypothalamic level to inhibit the release of ACTH and thus lower circulating levels of adrenal steroids, although its anti-emetic activity may not be related to adrenal suppression. It has been postulated that dexamethasone might act by inhibiting prostaglandin synthesis (Rich *et al.*, 1980); certainly indomethacin can substantially reduce the emesis evoked by radiation in dogs (Carpenter *et al.*, 1986) and iboprufen has proved useful in humans (Stryker *et al.*, 1979). Some cytotoxic drugs such as methotrexate and radiation cause increases in the blood-CSF permeability barriers (Livrea *et al.*, 1985). The well known action of dexamethasone on the blood–brain barrier may therefore be important in counteracting this effect and reducing the number of potentially emetic agents that would otherwise enter the CNS. Although the area postrema is classically outside the blood–brain barrier we cannot discount the fact that dexamethasone might have important actions on the vasculature of this area and again prevent passage of emetic agents from the circulation to the chemoreceptor trigger zone. Our study provides a basis for extending the use of $5HT_3$ antagonists to combination therapy in the clinical situation, where GR38032F and dexamethasone, an effective anti-emetic agent which has beneficial actions in 'nausea', together may prove useful to the numbers of patients refractory to GR38032F used alone.

We would like to thank the MOD (Procurement Executive) for financial support and Glaxo Group Research for the gift of GR38032F. We are grateful to Dr P.L.R. Andrews for comments on the manuscript.

### References

AAPRO, M.S. & ALBERTS, D.S. (1981). High dose dexamethasone for prevention of cisplatin-induced vomiting. *Cancer Chemother. Pharmacol.*, **7**, 11.

ALLAN, S.G., CORNBLEET, M.N., WARRINGTON, P.S., GOLLAND, I.M., LEONARD, R.C.F. & SMYTH, J.F. (1984). Dexamethasone and high dose metoclopramide: efficacy in controlling cisplatin-induced nausea and vomiting. *Br. Med. J.*, **289**, 878.

ANDREWS, P.L.R. & HAWTHORN, J. (1987). Evidence for an extra-abdominal site of action of the $5HT_3$ receptor antagonist BRL24924 in the inhibition of radiation-evoked emesis in the ferret. *Neuropharmacology*, **26**, 1367.

BERMUDEZ, J., BOYLE, E.A., MINER, W.D. & SANGER, G.J. (1988). The anti-emetic potential of the 5-hydroxytrypamine$_3$ receptor antagonist BRL43694. *Br. J. Cancer*, **58**, 644.

BOYLE, E.A., MINER, W.A. & SANGER, G.J. (1987) Anti-emetic activity of BRL43694, a novel $5HT_3$ receptor antagonist. *Br. J. Cancer*, **56**, 227.

BRITTAIN, R.T., BUTLER, A., COATES, I.H. & 11 others (1987). GR38032F a novel selective $5HT_3$ receptor antagonist. *Br. J. Pharmacol.*, **90**, 87P.

BRUERA, E.D., ROCA, E., CEDARO, L., CHACON, R. & ESTEVAZ, R. (1983). Improved control of chemotherapy-induced emesis by the addition of dexamethasone to metoclopramide in patients resistant to metoclopramide. *Cancer Treat. Rep.*, **67**, 381.

CARMICHAEL, J., CANTWELL, B.M.J., EDWARDS, C.M., RAPEPORT, W.G. & HARRIS, A.L. (1988). The serotonin type 3 receptor antagonist BRL43694 and nausea and vomiting induced by cisplatin. *Br. Med. J.*, **297**, 110.

CARPENTER, D.O., BRIGGS, D.B., KNOX, A.P. & STROMINGER, N.L. (1986) Radiation induced emesis in the dog: effects of lesions and drugs. *Radiat. Res.*, **108**, 307.

COATES, A., ABRAHAM, S., KAYE, S.B. & 4 others (1983). On the receiving end – patient perception of the side effects of cancer chemotherapy. *Eur. J. Cancer Clin. Oncol.*, **19**, 203.

COSTALL, B., DOMENEY, A.M., NAYLOR, R.J. & TATTERSALL, F.D. (1986). 5-hydroxytryptamine M-receptor antagonism to prevent cis-platin induced emesis. *Neuropharmacology*, **25**, 959.

CUNNINGHAM, D., HAWTHORN, J., POPLE, A. & 4 others (1987). Prevention of emesis in patients receiving cytotoxic drugs by GR38032F, a selective $5HT_3$ receptor antagonist. *Lancet*, **i**, 1461.

CUNNINGHAM, D., TURNER, A., HAWTHORN, J. & ROISIN, R.D. (1989). Ondansetron with and without dexamethasone to treat chemotherapy induced emesis. *Lancet*, **i**, 1323.

FAKE, C.S., KING, F.D. & SANGER, G.J. (1987). BRL43694: a potent and novel $5HT_3$ receptor antagonist. *Br. J. Pharmacol. Proc. Suppl.*, **91**, 335P.

FLORCZYCK, A.P., SCHURIG, J.E. & BRADNER, W.T. (1982). Cisplatin-induced emesis in the ferret: a new animal model. *Cancer Treat. Rep.*, **66**, 187.

FOZARD, J.R. (1984). Neuronal 5-HT receptors in the periphery. *Neuropharmacology*, **23**, 1473.

GRALLA, R.J. (1983). Metoclopramide: a review of anti-emetic trials. *Drugs*, **25** (suppl.) 63.

GRALLA, R.J., ITRI, L.M., PISKO, S.E. *et al.* (1981). Anti-emetic efficacy of high dose metoclopramide: randomized trials with placebo and prochlorperazine in patients with chemotherapy induced nausea and vomiting. *N. Engl. J. Med.*, **305**, 905.

GYLYS, J.A. & GIDDA, J.S. (1986). Rediation induced emesis in ferrets: an experimental model of emesis. *Gastroenterology*, **90**, 1446.

HAWTHORN, J. & ANDREWS, P.L.R. (1988). Can nausea be measured in animals? In *Proceedings of the symposium, Nausea and Vomiting a Multidisciplinary Perspective*, p. 31. Satellite Symposium of American Neuroscience Association: Ottawa.

HAWTHORN, J., OSTLER, K.J. & ANDREWS, P.L.R. (1988). The role of the abdominal visceral innervation and 5-HT-M receptors in vomiting induced by the cytotoxic drugs cyclophosphamide and cis-platin in the ferret. *Q. J. Physiol.*, **73**, 7.

KING, G.L. (1988). Characterisation of radiation induced emesis in the ferret. *Radiat. Res.*, **114**, 599.

LASZLO, J. (1983). Emesis as a limiting toxicity in cancer chemotherapy. In *Anti-emetics and Cancer Chemotherapy*, Lazlo, J. (ed) p. 1. Williams & Wilkins: Baltimore.

LEIBUNDGUT, U. & LANCRANJAN, I. (1987). First results with ICS 205–930 ($5HT_3$ receptor antagonist) in prevention of chemotherapy-induced emesis *Lancet*, **i**, 1198.

LIVREA, P., TROJANO, M., SIMONE, I.L. & 6 others (1985). Acute changes in blood-CSF barrier permselectivity to serum proteins after intrathecal methotrexate and CNS irradiation. *J. Neurol.*, **231**, 336.

MINER, W.D. & SANGER, G.J. (1968). Inhibition of cis-platin induced vomiting by selective 5-hydroxytryptamine M-receptor antagonism. *Br. J. Pharmacol.*, **88**, 497.

MINER, W.D., SANGER, G.J. & TURNER, D.H. (1986). Comparison of the effect of BRL24924, metoclopramide and domperidone on cis-platin induced emesis in the ferret. *Br. J. Pharmacol.*, **88**, 374P.

PALMER, M.C. & COLLS, B.M. (1987). Amelioration of cytotoxic-induced emesis with high-dose metoclopramide, dexamethasone and lorazepam. *Cancer Chemother. Pharmacol.*, **19**, 331.

POLLERA, C.F., NARDI, M., MAROLLA, P. & CARLINI, P. (1987). A randomized trial comparing alizapride alone or with dexamethasone vs a metoclopramide-dexamethasone combination for emesis induced by moderate dose cisplatin. *Cancer Chemother. Pharmacol.*, **19**, 335.

PREISTMAN, T., CHALLONER, T., BUTCHER, M. & PREISTMAN, S. (1988). Control of radiation induced emesis with GR38032F. *Proc. Am. Soc. Clin. Oncol.*, **7**, 281.

RICH, W.H., ABDULHAYOGLU, G. & DISAIA, P.J. (1980). Methylprednisolone as an anti-emetic during cancer chemotherapy – a pilot study. *Gynecol. Oncol.* **9**, 193.

RICHARDSON, B.P., ENGEL, G., DONATSCH, P. & STADLER, P.A. (1985). Identification of serotonin M-receptor subtypes and their specific blockade by a new class of drugs. *Nature*, **316**, 126.

STABLES, R., ANDREWS, P.L.R., BAILEY, H.E. & 5 others (1987). Anti-emetic properties of the $5HT_3$-receptor antagonist, GR38032F. *Cancer Treat. Rev.* **14**, 333.

STRYKER, J.A., DEMERS, L.M. & MORTEL, R. (1979). Prophylactic iboprufen administration during pelvic irradiation. *Int. J. Radiat. Oncol. Biol. Phys.*, **5**, 2049.

TUOR, U.I., KONDYSAR, M.H. & HARDING, R.K. (1988). Emesis radiation exposure, and local cerebral blood flow in the ferret. *Radiat. Res.* **114**, 532.


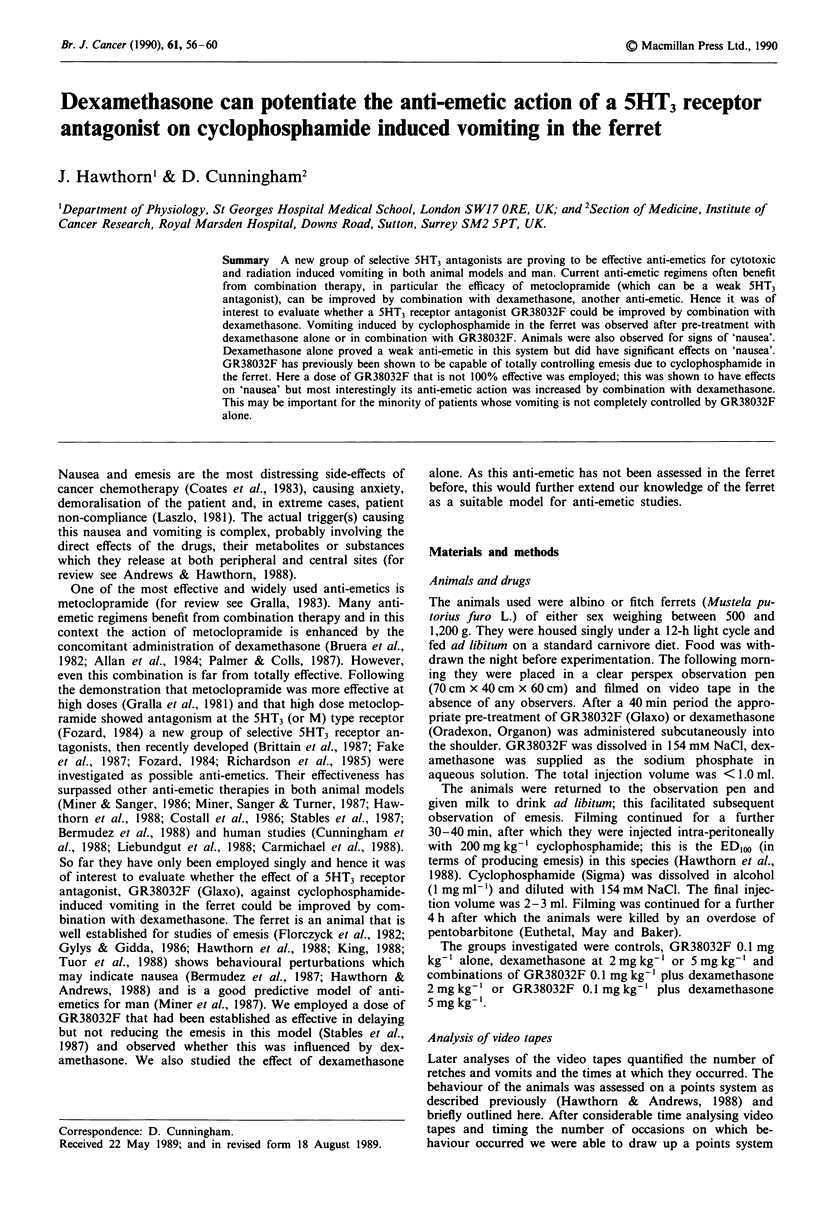

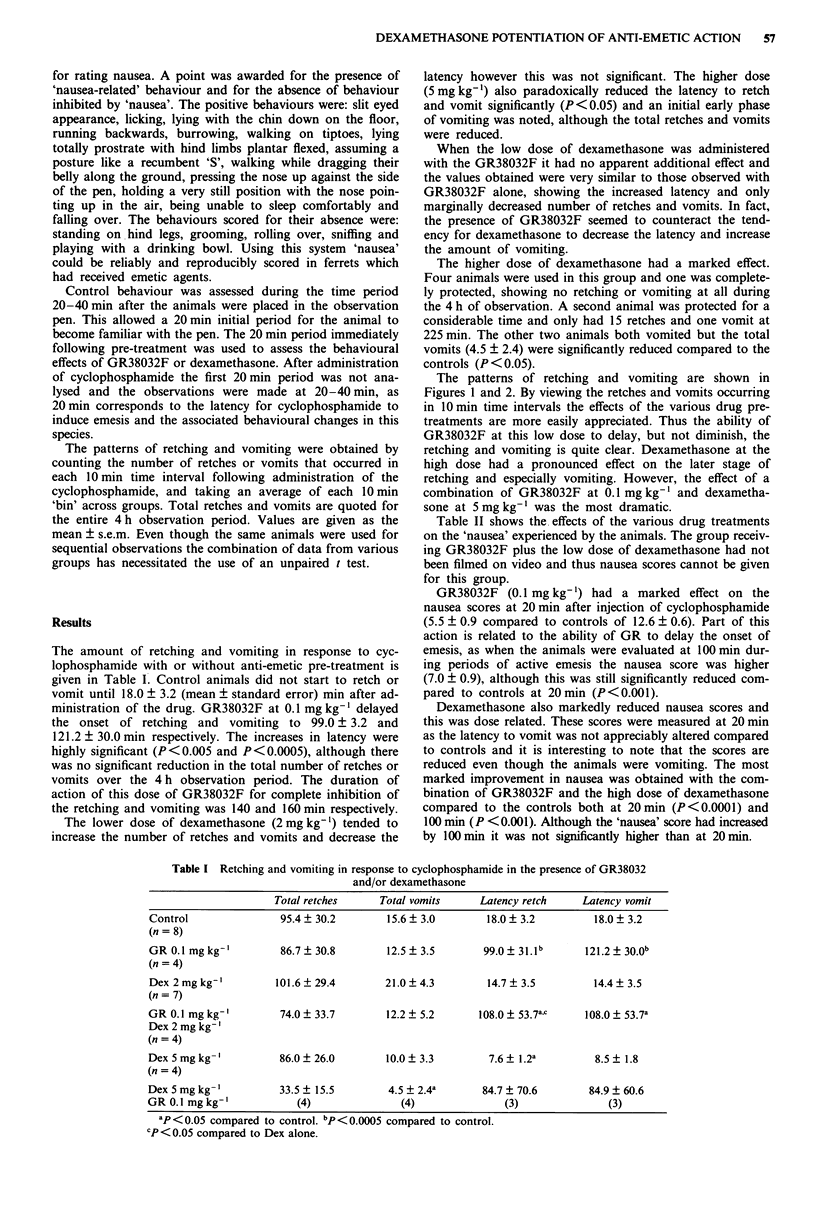

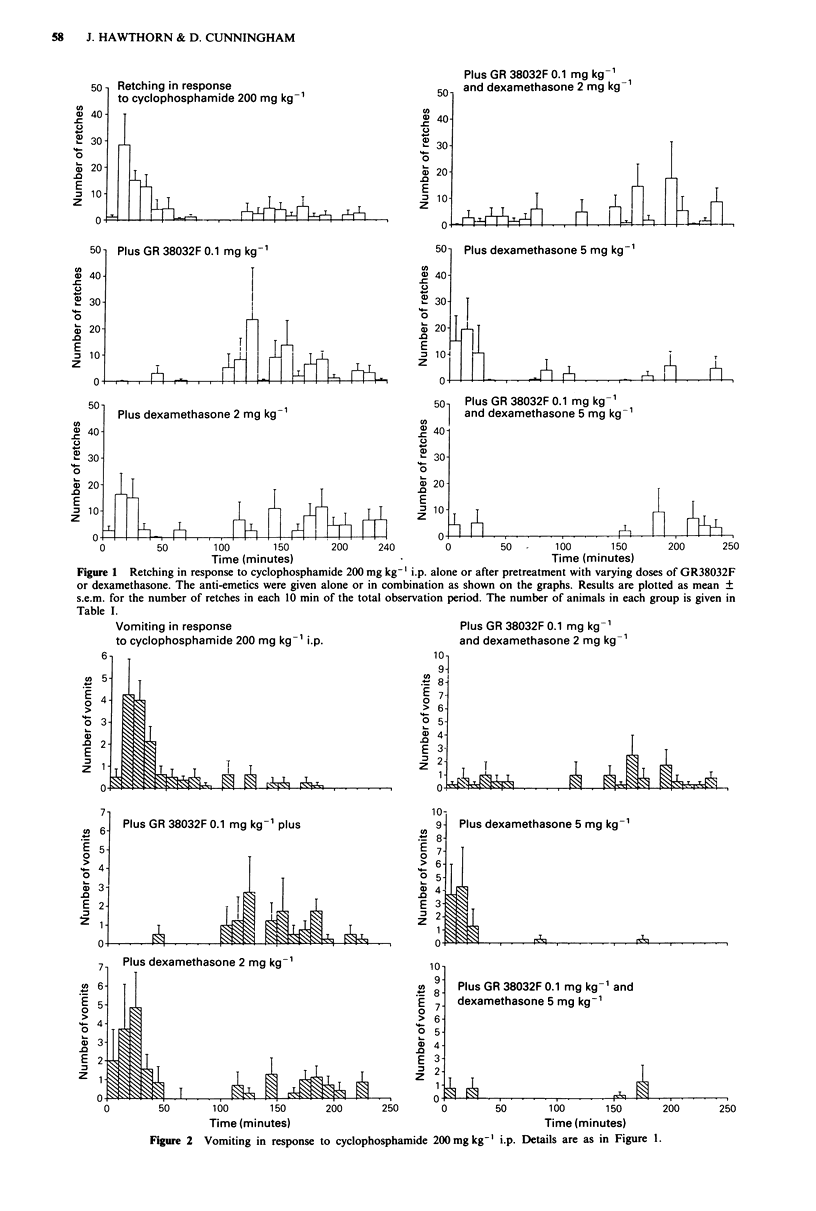

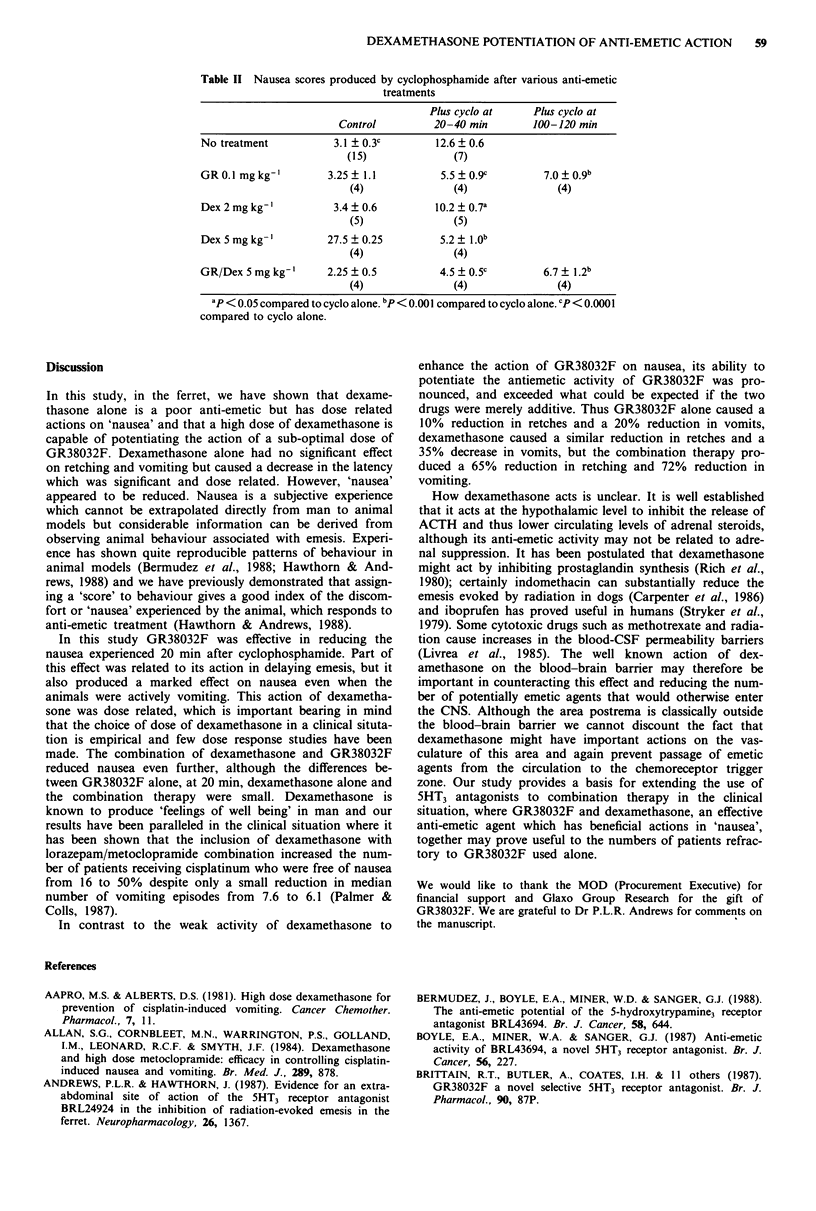

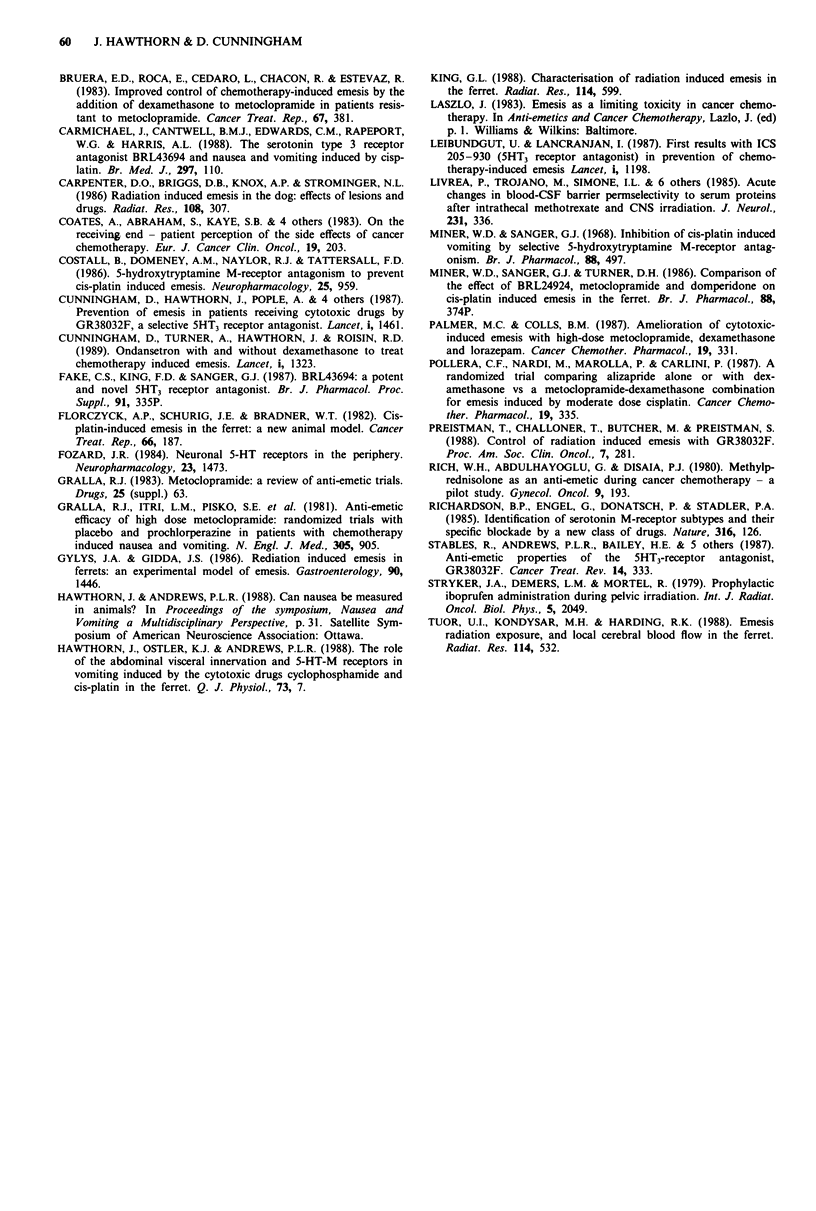


## References

[OCR_00676] Aapro M. S., Alberts D. S. (1981). High-dose dexamethasone for prevention of cis-platin-induced vomiting.. Cancer Chemother Pharmacol.

[OCR_00681] Allan S. G., Cornbleet M. A., Warrington P. S., Golland I. M., Leonard R. C., Smyth J. N. (1984). Dexamethasone and high dose metoclopramide: efficacy in controlling cisplatin induced nausea and vomiting.. Br Med J (Clin Res Ed).

[OCR_00687] Andrews P. L., Hawthorn J. (1987). Evidence for an extra-abdominal site of action for the 5-HT3 receptor antagonist BRL24924 in the inhibition of radiation-evoked emesis in the ferret.. Neuropharmacology.

[OCR_00693] Bermudez J., Boyle E. A., Miner W. D., Sanger G. J. (1988). The anti-emetic potential of the 5-hydroxytryptamine3 receptor antagonist BRL 43694.. Br J Cancer.

[OCR_00710] Bruera E. D., Roca E., Cedaro L., Chacón R., Estévez R. (1983). Improved control of chemotherapy-induced emesis by the addition of dexamethasone to metoclopramide in patients resistant to metoclopramide.. Cancer Treat Rep.

[OCR_00716] Carmichael J., Cantwell B. M., Edwards C. M., Rapeport W. G., Harris A. L. (1988). The serotonin type 3 receptor antagonist BRL 43694 and nausea and vomiting induced by cisplatin.. BMJ.

[OCR_00722] Carpenter D. O., Briggs D. B., Knox A. P., Strominger N. L. (1986). Radiation-induced emesis in the dog: effects of lesions and drugs.. Radiat Res.

[OCR_00727] Coates A., Abraham S., Kaye S. B., Sowerbutts T., Frewin C., Fox R. M., Tattersall M. H. (1983). On the receiving end--patient perception of the side-effects of cancer chemotherapy.. Eur J Cancer Clin Oncol.

[OCR_00732] Costall B., Domeney A. M., Naylor R. J., Tattersall F. D. (1986). 5-Hydroxytryptamine M-receptor antagonism to prevent cisplatin-induced emesis.. Neuropharmacology.

[OCR_00737] Cunningham D., Hawthorn J., Pople A., Gazet J. C., Ford H. T., Challoner T., Coombes R. C. (1987). Prevention of emesis in patients receiving cytotoxic drugs by GR38032F, a selective 5-HT3 receptor antagonist.. Lancet.

[OCR_00741] Cunningham D., Turner A., Hawthorn J., Rosin R. D. (1989). Ondansetron with and without dexamethasone to treat chemotherapy-induced emesis.. Lancet.

[OCR_00751] Florczyk A. P., Schurig J. E., Bradner W. T. (1982). Cisplatin-induced emesis in the Ferret: a new animal model.. Cancer Treat Rep.

[OCR_00756] Fozard J. R. (1984). Neuronal 5-HT receptors in the periphery.. Neuropharmacology.

[OCR_00764] Gralla R. J., Itri L. M., Pisko S. E., Squillante A. E., Kelsen D. P., Braun D. W., Bordin L. A., Braun T. J., Young C. W. (1981). Antiemetic efficacy of high-dose metoclopramide: randomized trials with placebo and prochlorperazine in patients with chemotherapy-induced nausea and vomiting.. N Engl J Med.

[OCR_00781] Hawthorn J., Ostler K. J., Andrews P. L. (1988). The role of the abdominal visceral innervation and 5-hydroxytryptamine M-receptors in vomiting induced by the cytotoxic drugs cyclophosphamide and cis-platin in the ferret.. Q J Exp Physiol.

[OCR_00787] King G. L. (1988). Characterization of radiation-induced emesis in the ferret.. Radiat Res.

[OCR_00796] Leibundgut U., Lancranjan I. (1987). First results with ICS 205-930 (5-HT3 receptor antagonist) in prevention of chemotherapy-induced emesis.. Lancet.

[OCR_00801] Livrea P., Trojano M., Simone I. L., Zimatore G. B., Logroscino G. C., Pisicchio L., Lojacono G., Colella R., Ceci A. (1985). Acute changes in blood-CSF barrier permselectivity to serum proteins after intrathecal methotrexate and CNS irradiation.. J Neurol.

[OCR_00807] Miner W. D., Sanger G. J. (1986). Inhibition of cisplatin-induced vomiting by selective 5-hydroxytryptamine M-receptor antagonism.. Br J Pharmacol.

[OCR_00818] Palmer M. C., Colls B. M. (1987). Amelioration of cytotoxic-induced emesis with high-dose metoclopramide, dexamethasone and lorazepam.. Cancer Chemother Pharmacol.

[OCR_00823] Pollera C. F., Nardi M., Marolla P., Carlini P. (1987). A randomized trial comparing alizapride alone or with dexamethasone vs a metoclopramide-dexamethasone combination for emesis induced by moderate-dose cisplatin.. Cancer Chemother Pharmacol.

[OCR_00835] Rich W. M., Abdulhayoglu G., DiSaia P. J. (1980). Methylprednisolone as an antiemetic during cancer chemotherapy--a pilot study.. Gynecol Oncol.

[OCR_00840] Richardson B. P., Engel G., Donatsch P., Stadler P. A. (1985). Identification of serotonin M-receptor subtypes and their specific blockade by a new class of drugs.. Nature.

[OCR_00845] Stables R., Andrews P. L., Bailey H. E., Costall B., Gunning S. J., Hawthorn J., Naylor R. J., Tyers M. B. (1987). Antiemetic properties of the 5HT3-receptor antagonist, GR38032F.. Cancer Treat Rev.

[OCR_00850] Stryker J. A., Demers L. M., Mortel R. (1979). Prophylactic ibuprofen administration during pelvic irradiation.. Int J Radiat Oncol Biol Phys.

